# Adult RSV hospitalizations in Spain: clinical characteristics and risk factors for ICU admission, prolonged stay, and mortality across six seasons (2016–2017 to 2021–2022)

**DOI:** 10.3389/fpubh.2026.1781582

**Published:** 2026-03-16

**Authors:** José-Manuel Ramos-Rincón, Maria Paz Ventero, Héctor Pinargote-Celorio, Silvia Otero-Rodriguez, Juan-Carlos Rodriguez-Diaz, José Sánchez-Payá, Esperanza Merino

**Affiliations:** 1 Internal Medicine Department, Dr. Balmis General University Hospital, Alicante Institute for Health and Biomedical Research (ISABIAL), Alicante, Spain; 2 School of Medicine, Miguel Hernandez University of Elche, Sant Joan d'Alacant, Spain; 3 Microbiology Service, Dr. Balmis General University Hospital, Alicante Institute for Health and Biomedical Research (ISABIAL), Alicante, Spain; 4 Infectious Diseases Unit, Dr. Balmis General University Hospital, Alicante Institute for Health and Biomedical Research (ISABIAL), Alicante, Spain; 5 Preventive Service, Dr. Balmis General University Hospital, Alicante Institute for Health and Biomedical Research (ISABIAL), Alicante, Spain

**Keywords:** epidemics, hospitalizations, intensive care unit, length of stay, mortality, respiratory syncytial virus, RSV, Spain

## Abstract

**Objective:**

To describe the clinical characteristics of adults hospitalized with respiratory syncytial virus (RSV) in Spain over six epidemiological seasons (2016–2017 to 2021–2022) and identify factors associated with ICU admission, prolonged length of stay, and in-hospital mortality (IHM).

**Methods:**

Retrospective, population-based study in adults (≥15 years) hospitalized for RSV and included in the Spanish hospital discharge database (containing all records of hospitalizations in Spain). Factors linked to ICU admission, prolonged length of stay, and IHM were assessed using multivariable logistic regression.

**Results:**

A total of 20,695 adults (56.5% women) were hospitalized with RSV. Case numbers increased until 2018–2019, then declined during the COVID-19 pandemic. Patients aged 15–64 years accounted for 20.9% of the sample; 65–79 years, 31.2%; and ≥80 years, 47.9%. Common comorbidities included hypertension (61.3%), diabetes (28.0%), and heart failure (26.1%). ICU admission (6.4% of cases) was independently associated with age 15–64 years (adjusted odds ratio [aOR] 5.04, 95% confidence interval [CI] 4.21–6.03), COVID-19 co-infection (aOR 5.06, 95% CI 2.88–8.87), and RSV pneumonia (aOR 2.19, 95% CI 1.48–3.27). IHM increased with age (15–64 years: 4.0%; 65–79 years: 6.2%, aOR 1.67, 95% CI 1.37–2.04; and ≥80 years: 9.5%, aOR 3.32, 95% CI 2.73–4.04). Other key risk factors were ICU admission (aOR 6.23, 95% CI 5.30–7.33), leukemia (aOR 3.31, 95% CI 2.11–4.59), and neoplasm (aOR 2.90, 95% CI 2.43–3.47).

**Conclusions:**

RSV represents a significant and growing burden among hospitalized adults, particularly in older individuals and those with underlying comorbidities, who experience worse outcomes and higher mortality. These findings underscore the importance of prevention strategies, including vaccination of adults aged ≥60 years and high-risk groups.

## 1 Introduction

Respiratory syncytial virus (RSV) is one of the leading causes of acute lower respiratory infections, causing severe disease in infants, young children, and older adults. It is also a major cause of bronchiolitis and pneumonia, especially in immunocompromised patients and transplant recipients. Well known for its winter outbreaks in temperate countries, RSV seasonality is nevertheless less marked than for influenza. Advances in diagnostic tools—now more accessible and reliable—have also brought recognition that RSV can significantly affect individuals regardless of age or immune status (1).

Several European multicenter studies have characterized the epidemiology and burden of RSV in adults. The RSV Consortium in Europe (RESCEU) conducted a large international prospective cohort study in community-dwelling adults aged ≥60 years across several European countries, finding annual RSV infection rates of 4–7%; most cases were mild, with very few hospitalizations or deaths (2–4). Additionally, a retrospective multicountry analysis of national hospital admission data from Denmark, England, Finland, the Netherlands, Scotland, and Spain (2016–2023) showed a sharp increase in RSV-associated hospitalizations in older adults, with the highest rates in those ≥85 years (4). The study also highlighted that RSV-coded hospitalizations significantly underestimate the true burden compared to laboratory-confirmed cases, emphasizing the need for improved surveillance and diagnostic practices (3, 4). A time series analysis across six European countries estimated that adults over 85 years have measurable rates of RSV-associated hospitalizations, though lower than in children (4, 5). Additionally, a pan-European modeling study estimated an average of 158,229 RSV-associated adult hospitalizations annually in the European Union, with 92% occurring in those ≥65 years (5, 6).

In France, data reflect a high disease burden for RSV in adults, especially those over 75 and with respiratory or cardiac comorbidities. In these groups, RSV-related hospitalizations are associated with high morbidity, mortality, and admissions to the intensive care unit (ICU), with worse outcomes in the event of co-infections, particularly bacterial. The high rates of readmissions and death within 60 days of discharge further underline the need for preventive strategies in high-risk populations (7, 8).

In Spain, most studies of RSV are in children. Using a hospital discharge database to estimate the burden of RSV-related hospitalizations, Martinon-Torres et al. (9) highlighted the clinical and economic impact of RSV in infants aged under one year, while Heppe-Montero et al. (10) reported on the burden in both pediatric and older populations, with elevated rates of hospitalization and in-hospital mortality.

Fortunately, the RSV vaccine landscape has been advancing. Until 2023, no RSV vaccines were available for adults. However, three vaccines—GSK's Arexvy, Pfizer's Abrysvo, and Moderna's mRNA-1345—have now been approved in Spain for use in older adults, with EU indications extending to some younger age groups depending on the vaccine, though in Spain, they are not yet reimbursed through the national health system (11). This changing scenario reinforces the need for more robust data on RSV's impact in older adults.

In this study, we analyze six epidemiological seasons (2016–2017 to 2021–2022) of adult RSV-related hospitalizations in Spain, aiming to: (a) describe the clinical characteristics by age group; and (b) identify factors associated with ICU admission, length of hospital stay (LOS), and in-hospital mortality (IHM).

## 2 Materials and methods

### 2.1 Data sources

Data on hospital admissions were obtained from the Spanish National Surveillance System for Hospital Data (SNSSHD), specifically the Hospital Care Activity Record—Minimum Basic Data Set (Registro de Actividades Especializadas-Conjunto Mínimo Básico de Datos, RAE-CMBD), which contains all records of hospitalizations in all public and private hospitals in Spain. Diagnoses are coded using the International Classification of Diseases, 10th revision (ICD-10) (12).

### 2.2 Study population and variables

Inclusion criteria were: hospitalizations in adults (≥15 years) with RSV during the epidemiological season (week 40 of the first year to week 20 of the second), from 2016–2017 to 2021–2022. RSV-related hospitalization was defined as all recorded hospital admissions with a diagnosis of RSV, identified by ICD-10 codes: B97.4 (RSV as the cause of diseases classified elsewhere), J12.1 (pneumonia due to RSV), J20.5 (acute bronchitis due to RSV), and J21.0 (acute bronchiolitis due to RSV), as either a primary or secondary diagnosis. Laboratory confirmation for RSV was not available, but admissions with ICD-10 codes usually had molecular confirmation.

The variables collected for each hospitalization episode were age, sex, comorbidities, LOS, admission to the ICU, and IHM. Comorbidities were identified during the episode of hospitalization, not from past medical history were extracted using ICD-10 diagnostic codes (up to 20 diagnoses, Supplementary Table 1) and classified into the following categories: hypertensive disease (hypertension, cardiac hypertensive disease, kidney hypertensive disease, cardiac and kidney hypertensive disease), diabetes mellitus (type 1 and 2), chronic obstructive pulmonary disease (COPD), neurodegenerative disease (including dementia), chronic heart failure, chronic kidney disease, obesity, neoplasia, lymphoma, leukemia, HIV infection, cirrhosis, transplantation, and COVID-19 coinfection. The classification of ICU admission encompasses both patients admitted directly to the ICU upon hospitalization and those transferred to the ICU from a different ward. Hospital stays longer than 60 days were excluded from the analysis to reduce bias by removing atypical cases that likely had complications not directly related to RSV, minimizing confounders, and maintaining sample homogeneity. Outcome variables were: (1) ICU admission, (2) prolonged LOS (greater than the value of the 75th percentile (>11 days) as a cutoff), and (3) IHM.

### 2.3 Analysis

Cases were stratified by age group (15–64, 65–79, and ≥80 years), sex assigned at birth, and epidemiological season. Non-parametric continuous variables (as assessed by the one-sample Kolmogorov–Smirnov test) are expressed as medians and interquartile ranges (IQR), while categorical variables are expressed as absolute values and percentages. Bivariable comparisons of quantitative and categorical variables were performed using the Kruskal–Wallis test for quantitative variables and the chi-squared test for categorical variables. *P*-values of less than 0.05 were considered statistically significant.

Multivariable logistic regression analysis was used to identify independent predictors of ICU admission, prolonged LOS (>11 days), and IHM. The model included sex, age group, epidemiological season, and other variables yielding a *p*-value of less than 0.05 in the crude analysis, which were entered using a stepwise selection method with the likelihood ratio test. Model fit was evaluated using the area under the receiver operating characteristic curve (AUC) with 95% confidence intervals (CIs). The regression analysis values were expressed as adjusted odds ratios (aORs) and 95% CIs. All statistical analyses were performed using the IBM SPSS package for Windows v25.0 (IBM Corp, Armonk, NY).

### 2.4 Ethical aspects

The anonymized dataset was provided by the Spanish Ministry of Health upon request. According to Spanish legislation, informed consent was not required for studies using anonymized administrative databases. The research protocol was approved by the Ethics and Research Integrity Committee of Miguel Hernández University (Ref. AUT.DMC.JMRR.241103). The study adhered to the ethical principles of the revised Declaration of Helsinki (2013).

## 3 Results

### 3.1 Characteristics of adults hospitalized with RSV

Over six epidemiological seasons, a total of 91,367 patients with RSV infection were hospitalized in Spain. Of these, 77.3% were under 15 years old, while the remaining 22.7% of adults (*n* = 20,695, 56.5% female) comprised the final study sample. Patients had a median age of 79 years (IQR 67–86), and the distribution by age group was: 15–64 years, 20.9%; 65–79 years, 31.2%; and ≥80 years, 47.9%. Most cases (69.2%) were coded as RSV infection not otherwise specified. The most common comorbidities were hypertensive disease (61.3%), diabetes mellitus (28.0%), chronic heart failure (26.1%), chronic kidney disease (20.1%) and COPD (18.3%; Table 1).

**Table 1:** Characteristics of adults hospitalized with RSV infection by age group.

**Variables**	**Total (*N* = 20,695) % (*N*)**	**15–64 (*N* = 4,326, 20.9%) % (*N*)**	**65–79 (*N* = 6,649, 31.2%) % (*N*)**	**≥80 (*N* = 9,920, 47.9%) % (*N*)**	*P*-value^*^
**Sex**					
Male	43.5 (8,999)	51.3 (2,220)	51.4 (3,314)	34.9 (3,465)	<0.001
Female	56.5 (11,696)	48.7 (2,106)	48.6 (3,135)	65.1 (6,455)	
**Epidemiological season**					
2016–2017	12.8 (2,658)	14.5 (627)	12.6 (813)	12.3 (1,218)	<0.001
2017–2018	19.6 (4,053)	19.8 (857)	19.8 (1,279)	19.4 (1,927)	
2018–2019	29.4 (6,089)	27.2 (1,178)	29.4 (1,893)	30.4 (3,018)	
2019–2020	25.3 (5,227)	23.1 (999)	26.6 (1,715)	25.3 (1,513)	
2020–2021	0.2 (50)	0.3 (13)	0.3 (20)	0.2 (17)	
2021–2022	12.6 (2,608)	15.1 (652)	11.3 (729)	12.4 (1,227)	
**Type of RSV** ^†^					
RSV infection not otherwise specified	69.2 (14,322)	70.5 (3,049)	67.7 (4,459)	67.1 (6,714)	<0.001
RSV pneumonia	14.6 (3,023)	6.7 (723)	14.2 (917)	13.9 (1,553)	<0.001
RSV bronchitis	13.5 (2,790)	9.8 (426)	12.6 (811)	15.7 (1,553)	<0.001
RSV bronchiolitis	2.7 (560)	3.0 (128)	2.5 (162)	2.7 (270)	0.075
**Clinical presentation**					
Bronchospasm	20.1 (4,153)	7.8 (339)	17.5 (1,229)	27.1 (2,685)	<0.001
**Comorbidities**					
Hypertensive disease	61.3 (2,682)	28.6 (1,238)	62.8 (4,052)	74.5 (7,392)	<0.001
Diabetes mellitus	28.0 (5,793)	16.0 (690)	32.3 (2,986)	30.4 (3,017)	<0.001
Chronic heart failure	26.1 (5,394)	7.3 (316)	21.6 (1,392)	37.2 (3,686)	<0.001
Chronic kidney failure	20.1 (4,153)	7.8 (339)	17.5 (1,129)	27.1 (2,685)	<0.001
COPD	18.3 (3,783)	15.8 (682)	24.3 (1,569)	15.4 (1,532)	<0.001
Obesity	12.2 (2,524)	13.8 (598)	14.7 (946)	9.9 (980)	<0.001
Ischemic heart disease	11.5 (2,382)	5.3 (231)	13.2 (852)	13.1 (1,299)	<0.001
Asthma	10.4 (2,149)	12.3 (530)	10.8 (696)	9.3 (923)	<0.001
Neurodegenerative disease	9.3 (1,924)	0.9 (40)	4.1 (264)	16.3 (1,620)	<0.001
Solid neoplasia	6.2 (1,286)	8.1 (350)	8.0 (514)	4.3 (422)	<0.001
Lymphoma	5.7 (1,189)	456 (10.5)	493 (7.6)	240 (2.4)	<0.001
Transplantation	3.4 (699)	422 (9.8)	249 (3.9)	28 (0.3)	<0.001
Leukemia	1.3 (277)	3.2 (140)	1.5 (99)	0.4 (38)	<0.001
Cirrhosis	1.2 (242)	2.3 (99)	1.6 (101)	0.4 (42)	<0.001
HIV infection	1.0 (198)	4.3 (184)	0.2 (13)	0.0 (1)	<0.001
COVID-19	0.5 (104)	0.7 (31)	0.5 (31)	0.4 (42)	0.072
**Outcome**					
Length of hospital stay (days), ^‡^median (IQR)	7 (4–11)	7 (4–12)	7 (5–12)	7 (5–11)	<0.001
ICU admission	6.4 (1,306)	12.0 (515)	9.0 (576)	2.2 (215)	<0.001
In-hospital death	7.3 (1,519)	4.0 (172)	6.2 (401)	9.5 (946)	<0.001

COPD: chronic obstructive pulmonary disease; ICU: intensive care unit; IQR: interquartile range; RSV: respiratory syncytial virus.

^*^Qualitative variables were analyzed using the chi-squared test, and continuous variable by the Kruskal-Wallis test.

^†^RSV infection not otherwise specified = B97.4 (respiratory syncytial virus as the cause of diseases classified elsewhere); RSV pneumonia = J12.1 (pneumonia due to respiratory syncytial virus), RSV bronchitis = J20.5 (Acute bronchitis due to respiratory syncytial virus), RSV bronchiolitis = J21.0 (Acute bronchiolitis due to respiratory syncytial virus).

^‡^Hospitalizations exceeding 60 days were excluded from this analysis, available data was for 20,505 patients.

The number of cases increased from 2,658 in the 2016–2017 season to 6,089 in 2018–2019 and 5,227 in 2019–2020. However, the 2020–2021 season, characterized by the dominance of the COVID-19 pandemic, saw a sharp drop in RSV hospitalizations, to just 50 cases, before rising again to 2,608 in 2021–2022.

Table 1 shows the characteristics of hospitalizations by age group. The relative proportion of women increases significantly with the age, as does the rate of RSV pneumonia, RSV bronchitis, bronchospasm, and several comorbidities: hypertensive disease, diabetes mellitus, chronic heart failure, chronic kidney failure, and ischemic heart disease. The prevalence of other comorbidities decreased with age: COPD, obesity, asthma, neoplasm, lymphoma, transplantation, leukemia, cirrhosis, and HIV infection.

### 3.2 ICU admissions

Altogether, 6.4% of the 20,466 cases with available data on ICU admissions presented this outcome (Table 2). The proportion of ICU admissions in patients with RSV pneumonia was higher (11.0%) than for those with RSV-acute bronchitis (4.2%) and RSV-acute bronchiolitis (5.7%; *p* < 0.001). ICU admission was also more frequent in patients under 65 years old (12.6%) than in older people (65–79 years, 9.0%) and especially compared to very old people (≥80 years, 2.2%) (*p* < 0.001). The highest ICU admission rate was observed during the 2020–2021 season (COVID-19 pandemic), at 12.0%, while the lowest was in the 2021–2022 season (5.0%). IHM among ICU-admitted patients was 21.7%, significantly higher than the 6.4% observed in non-ICU patients (*p* < 0.001).

**Table 2:** Crude and adjusted^*^ odds ratios for risk factors associated with ICU admission in patients with RSV infection.

**Variables**	**ICU admission % (*n*/*N*)^†^**	Crude analysis		Multivariable analysis	
		**OR (95% CI)**	***P*** **-value**	**aOR (95% CI)** ^*^	***P*** **-value** ^‡^
**Sex**					
Male	7.8 (697/8,893)	1.53 (1.37–1.71)	<0.001	1.14 (1.01–1.29)	0.028
Female	5.3 (609/11,573)	1		1	
**Age group (year)**					
15–64	12.0 (515/4,285)	6.09 (5.17–7.17)	<0.001	5.04 (4.21–6.03)	<0.001
65–79	9.0 (575/6,380)	4.42 (3.71–5.19)	<0.001	3.78 (3.21–4.46)	<0.001
≥80	2.2 (215/9,801)	1	—	1	—
**Epidemiological season**					
2016–2017	7.2 (179/2,473)	1.49 (1.49–1.89)	0.001	1.78 (1.39–2.29)	<0.001
2017–2018	6.1 (248/4,025)	1.25 (1.00–1.55)	0.044	1.55 (1.23–1.96)	<0.001
2018–2019	6.3 (383/6,089)	1.28 (1.04–1.58)	0.016	1.67 (1.34–2.09)	<0.001
2019–2020	6.9 (362/5,227)	1.42 (1.16–1.75)	0.001	1.80 (1.44–2.52)	<0.001
2020–2021	12.0 (6/50)	2.61 (1.94–6.24)	0.031	1.46 (0.55–3.87)	0.45
2021–2022	5.0 (128/2,582)	1		1	
**Type of RSV**					
RSV infection not otherwise specified	5.8 (828/14,159)	1.03 (0.71–1.49)	0.85	1.05 (0.74–1.53)	0.78
RSV pneumonia	11.0 (330/3,003)	2.06 (1.40–3.01)	<0.001	2.19 (1.48–3.27)	<0.001
RSV bronchitis	4.2 (117/2,756)	0.73 (0.49–1.11)	0.15	0.86 (0.75–0.87)	0.49
RSV bronchiolitis	5.7 (31/548)	1		1	-
**Hypertensive disease**					
No	8.3 (653/7,914)	1		1	
Yes	5.2 (652/12,552)	0.61 (0.54–0.83)	0.001	0.85 (0.75–0.98)	0.021
**Diabetes mellitus**					
No	6.4 (943/14,733)	1		NI	—
Yes	6.3 (363/5,733)	0.98 (0.87–1.12)	0.52	NI	—
**Chronic heart failure**					
No	6.6 (993/15,138)			NI	—
Yes	5.9 (313/5,328)	0.89 (0.78–1.14)	0.079	NI	—
**Chronic kidney failure**					
No	6.7 (1,099/16,358)	1		1	
Yes	5.0 (207/4,108)	0.73 (0.63–0.86)	<0.001	1.02 (0.89–1.20)	0.82
**COPD**					
No	6.0 (10,002/16,728)	1	—	1	—
Yes	8.1 (304/3,738)	1.38 (1.21–1.58)	<0.001	1.27 (1.10–1.46)	0.001
**Obesity**					
No	6.1 (1,092/17,968)		—	1	—
Yes	8.6 (214/2,498)	1.44 (1.24/1.68)	<0.001	1.33 (1.13–1.57)	<0.001
**Ischemic heart disease**					
No	6.2 (1,128/18,103)	1	—	1	—
Yes	7.5 (178/2,363)	1.22 (1.04–1.44)	0.017	1.43 (1.20–1.78)	<0.001
**Asthma**					
No	6.4 (1,179/18,339)	1		NI	
Yes	6.0 (127/2,127)	0.94 (0.76–1.11)	0.41	NI	—
**Neurodegenerative disease**					
No	6.9 (1,286/18,556)	1	—	1	
Yes	1.0 (20/1,910)	0,14 (0.09–0.22)	<0.001	0,27 (0.76–4.35)	<0.001
**Solid neoplasia**					
No	6.5 (1,244/19,188)	1			
Yes	4.9 (62/1,278)	0.73 (0.57–0.95)	0.023	0.56 (0.43–0.74)	<0.001
**Lymphoma**					
No	6.4 (1,229/19,296)	1	—	NA	
Yes	6.6 (77/1,170)	1.03 (0.81–1.31)	0.77	NA	—
**Transplantation**					
No	6.4 (1,269/19,775)	1	—	NA	
Yes	5.4 (37/691)	0.82 (0.59–1.15)	0.38	NA	—
**Leukemia**					
No	6.3 (1,278/20,190)	1	—	1	
Yes	10.1 (28/276)	1.67 (1.12–2.48)	0.014	1.08 (0.72–1.62)	0.69
**Cirrhosis**					
No	6.3 (1,282/20,225)	1	—	1	—
Yes	10.0 (24/241)	1.63 (1.07–2.50)	0.03	1.12 (0.72–1.73)	0.61
**HIV infection**					
No	6.3 (1,281/20,269)	1	—	1	—
Yes	12,7 (25/197)	2.15 (1.41–3.29)	<0.001	1.09 (0.70–1.68)	0.69
**COVID-19**					
No	6.3 (1,285/20,363)	1	—	1	—
Yes	20.4 (21/103)	3.80 (2.34–6.16)	<0.001	5.06 (2.88–8.87)	<0.001

aOR, adjusted odds ratio; CI, confidence interval; COPD, chronic obstructive pulmonary disease; ICU, intensive care unit; NA, not applicable (not included in model); OR, odds ratio; RSV, respiratory syncytial virus.

^*^Multivariable logistic regression analysis was used to identify independent predictors of ICU admission; model included variables yielding a p-value of less than 0.05 in the crude analysis, plus sex, group of age, and epidemiological season.

^†^The results are presented as percentages (number of events/sample size).

^‡^Adjusted odds ratios were calculated using a multivariate analysis that included age group, epidemiological season, and variables with a p-value <0.05 in the univariate analysis.

After multivariable analysis adjusted for the epidemiological season, factors associated with ICU admission included COVID-19 coinfection, age, RSV pneumonia, ischemic heart disease, obesity, COPD, and male sex. Conversely, factors associated with a lower likelihood of ICU admission were neurodegenerative disease and neoplasm (Table 2).

### 3.3 Length of stay

After excluding hospitalizations longer than 60 days, the median LOS in both the overall sample and in those not admitted to the ICU was 7 days (IQR 4–11); for those in intensive care, median LOS was more than twice as long (15 days, IQR 9–24; *p* < 0.001). Among patients who died during admission, the median stay was 8 days (IQR 4–16), compared to 7 days (IQR 4–11) for survivors (*p* < 0.001).

The total number of hospital admission days was 193,873, concentrated in the 2018–2019 epidemiological season. Overall, 44.8% of total hospital days corresponded to patients aged over 80 years. Prolonged LOS, as determined using the cutoff of the 75th percentile, was defined as 11 days or longer in our study; Table 3 summarizes the epidemiological and clinical characteristics of these patients.

**Table 3:** Crude and adjusted odds ratios^*^ for conditions associated with prolonged length of stay (>11 days) in adults hospitalized with RSV infection.

**Variables**	**Length of stay >11 days % (*n*/*N*)^†^**	Crude analysis		Multivariable analysis	
		**OR (95% CI)**	***P*** **-value**	**aOR (95% CI)** ^*^	***P*** **-value** ^‡^
**Sex**					
Male	25.0 (2,220/8,891)	1		1	0.149
Female	24.0 (1,794/11,627)	0.95 (0.89–1.01)		0.95 (0.88–1.02)	
**Age group (years)**					
15–64	25.6 (1,089/4,252)	1.17 (1.09–1.27)	<0.001	1.03 (0.93–1.13)	0.61
65–79	26.4 (1,684/6,373)	1.22 (1.14–1.31)	<0.001	1.10 (1.02–1.19)	0.018
≥80	22.7 (2,241/9,892)	1	1	1	
**Epidemiological season**					
2016–2017	26.2 (687/2,621)	1.16 (1.02–1.31)	0.020	1.13 (0.99–1.32)	0.64
2017–2018	25.0 (1,008/4,025)	1.09 (0.97–1.22)	0.14	1.10 (0.73–1.30)	0.13
2018–2019	24.0 (1,452/6,038)	1.03 (0.92–1.15)	0.54	1.03 (0.91–1.15)	0.64
2019–2020	24.0 (5,195/6,195)	1.03 (0.92–1.15)	0.57	0.99 (0.88–1.11)	0.92
2020–2021	25.0 (12/48)	1.08 (0.56–2.10)	0.80	0.75 (0.37–1.54)	0.45
2021–2022	23.4 (607/2,560)	1	—	1	—
**Type of RSV**					
RSV infection not otherwise specified	24.8 (3,516/14,193)	1.25 (0.99–1.50)	0.054	1.24 (1.00–1.54)	0.047
RSV pneumonia	26.9 (804/2,990)	1.36 (1.10–1.70)	0.005	1.24 (0.99–1.56)	0.060
RSV bronchitis	20.7 (576/2,777)	0.97 (0.78–1.21)	0.81	1.03 (0.82–1.20)	0.77
RSV bronchiolitis	21.2 (110/557)	1	—	1	—
**Comorbidities**					
**Hypertensive disease**					
No	24.4 (1,929/7,916)	1	-	NI	—
Yes	24.5 (3,085/12,601)	1.00 (0.94–1.07)	0.86	NI	—
**Diabetes mellitus**					
No	23.9 (3,531/14,757)	1	0.06	NI	—
Yes	25.7 (1,483/5,760)	1.10 (1.02–1.18)		NI	—
**Chronic heart failure**					
No	22.5 (3,407–15,168)	1	—	—	—
Yes	30.0 (1,607/5,349)	1.48 (1.38–1.58)	<0.001	1.59 (1.47–1.72)	<0.001
**Chronic kidney failure**					
No	23.5 (3,856/16,398)	1	—	1	—
Yes	28.1 (1,148/4,119)	1.17 (1.17–1.37)	<0.001	1.27 (1.16–1.38)	<0.001
**COPD**					
No	24.4 (4,084/16,769)	1	—	NI	—
Yes	24.8 (930/3,780)	1.05 (0.94–1.11)	0.535	NI	—
**Obesity**					
No	24.1 (4,361/18,005)	1	—	1	—
Yes	26.8 (673/2,512)	1.15 (1.05–1.26)	0.003	1.09 (0.99–1.21)	0.078
**Ischemic heart disease**					
No	24.1 (4,376/18,146)	1	—	1	—
Yes	26.8 (626/2,371)	1.15 (1.05–1.27)	0.004	0.96 (0.86–1.06)	0.42
No	24.7 (4,533/18,300)	1	—	1	—
Yes	22.5 (481/2,137)	0.88 (0.79–0.98)	0.030	0.97 (0.87–1.09)	0.57
**Neurodegenerative disease**					
No	24.7 (4,594/18,597)	1	—	1	—
Yes	21.9 (420/1,920)	0.85 (0.76–0.85)	0.006	0.95 (0.84–1.07)	0.419
**Solid neoplasia**					
No	24.0 (4,618/19,243)	1	—	1	—
Yes	31.1 (396/1,274)	1.42 (1.26–1.61)	<0.001	1.63 (1.43–1.86)	<0.001
**Lymphoma**					
No	24.0 (4,647/19,351)	1	—	1	—
Yes	31.5 (367/1,166)	1.45 (1.27–1.88)	<0.001	1.68 (1.47–1.93)	<0.001
**Transplantation**					
No	24.4 (4,929/19,824)	1	—	NI	—
Yes	26.4 (185/693)	1.13 (0.95–1.34)	0.159	NI	—
**Leukemia**					
No	24.1 (4,886/20,251)	1		1	—
Yes	48.1 (128/266)	2.91 (2–29–3.71)	<0.001	3.30 (2.64–4.40)	<0.001
**Cirrhosis**					
No	24.3 (4,932/20,281)	1	—	1	—
Yes	34.7 (82/236)	1.65 (1.26–2.17)	<0.001	1.59 (1.20–2.12)	0.001
**HIV infection**					
No	24.4 (3,951/20,322)	1	—	1	—
Yes	32.3 (63/195)	1.43 (1–09–2.00)	0.011	1.59 (1.14–2.20)	0.005
**COVID-19**					
No	24.3 (4,971/20,420)	1	—	—	—
Yes	44.3 (43/97)	2.45 (1.65–3.69)	<0.001	2.22 (1.50–3,64)	<0.001
**ICU admission**					
No	21.9 (4,179/19,059)	1	—	1	—
Yes	64.0 (788/1,231)	6.33 (5.63–7.15)	<0.001	6.26 (5.51–7.11)	<0.001
**In-hospital death**					
No	23.5 (4,467/19,015)	1	—	1	—
Yes	35.6 (543/1,490)	1.88 (1.67–2.08)	<0.001	1.33 (1.18–1.50)	<0.001

aOR: adjusted odds ratio; CI: confidence interval; COPD: chronic obstructive pulmonary disease; ICU: intensive care unit; NA: not applicable (not included in model); OR: odds ratio; RSV: respiratory syncytial virus.

^*^Multivariable logistic regression analysis was used to identify independent predictors of ICU admission; model included variables yielding a p-value of less than 0.05 in the crude analysis, plus sex, group of age, and epidemiological season.

^†^The results are presented as percentages (number of events/sample size).

^‡^Adjusted odds ratios were calculated using a multivariate analysis that included age group, epidemiological season, and variables with a p-value <0.05 in the univariate analysis.

In the multivariable analysis—adjusted for epidemiological season, sex, age group, and variables with a *p*-value under 0.05—the factors independently associated with prolonged LOS included ICU admission, leukemia, and COVID-19 co-infection. Several chronic conditions were also linked to longer stays, such as lymphoma, neoplasms, chronic heart disease, chronic kidney failure, cirrhosis, and HIV infection. No significant associations were observed with sex, age, epidemiological season, hypertension, diabetes mellitus, COPD, obesity, asthma, neurodegenerative diseases, or transplantation.

### 3.4 In-hospital mortality

Overall, 7.4% of hospitalized patients died. The case fatality rate increased with age: 4% in patients under 65 years, 6.2% in patients aged 65–79 years, and 9.5% in those aged over 80 years (*p* < 0.001). Among patients admitted to the ICU, the case fatality rate was 21.7% and increased with age, from 15.9% in those under 65 years to 29.3% in the oldest patients (*p* < 0.001). Individuals aged over 80 years accounted for 62.5% of total deaths. Among patients younger than 65 years, 48.5% of deaths occurred after ICU admission, compared to 6.3% in the oldest patients (*p* < 0.001). The overall mortality rate among hospitalized patients varied by epidemiological season, from 6.1% in the 2016–2017 season to 18.0% during the COVID pandemic (Table 4).

**Table 4:** Crude and adjusted odds ratios^*^ for risk factors associated with in-hospital mortality in adults hospitalized with RSV infection.

**Variables**	**Hospital mortality % (*n*/*N*) ^†^**	Crude analysis		Multivariable analysis	
		**OR (95% CI)**	***P*** **-value**	**aOR (95% CI)** ^*^	***P*** **-value** ^‡^
**Sex**					
Male	7.9 (710/8,996)	1.15 (1.03–1.27)	<0.001	1.11 (0.9–1.25)	0.066
Female	6.9 (808/11,686)	1	—	1	—
**Age group (year)**					
15–64	4.0 (172/4,323)	1	—	1	—
65–79	6.2 (401/6,443)	1.60 (1.34–1.92)	<0.001	1.67 (1.37–2.04)	<0.001
≥80	9.5 (946/9,916)	2.54 (2.15–3.00)	<0.001	3.32 (2.73–4.04)	<0.001
**Epidemiological season**					
2016–2017	6.1 (161/2,656)	1	—	1	—
2,017–2018	7.3 (291/4,063)	1.19 (0.98–1.45)	0.079	1.96 (0.97–1.47)	0.093
2018–2019	7.2 (435/6,083)	1.19 (0.99–1.43)	0.063	1.16 (0.95–1.14)	0.14
2019–2020	8.1 (423/5,225)	1.35 (1.13–1.54)	0.001	1.29 (1.06.-1.57)	0.012
2020–2021	18.0 (9/50)	3.04 (1.62–7.12)	0.001	3.10 (1.39–6.89)	0.005
2021–2022	7.7 (200/2,605)	1.28 (1.03–1.59)	0.021	1.31 (1.04–1.64)	0.020
**Type of RSV**					
RSV infection not otherwise specified	6.9 (993/14,311)	1.53 (0.81–1.64)	0.43	1.14 (0.79–1.63)	0.47
RSV pneumonia	11.0 (331/3,021)	1.90 (1.32–2.74)	0.001	1.66 (1.14–2.43)	0.008
RSV bronchitis	5.8 (161/2,790)	0.94 (0.64–1.38)	0.78	0.91 (0.61.1.35)	0.70
RSV bronchiolitis	6.1 (34/560)	1	—	1	—
**Hypertensive disease**					
No	7.2 (576/8,010)	1	—	NA	—
Yes	7.4 (943/12,672)	1.03 (0.93–1.56)	0.51	NA	—
**Diabetes mellitus**					
No	7.3 (1,088/14,889)	1	—	NA	
Yes	7,4 (431/5,793)	1.02 (0.90–1.14)	0.74	NA	—
**Chronic heart failure**					
No	6.4 (973/15,292)	1	—	1	—
Yes	10.1 (546/5,390)	1.69 (1.48–1.85)	<0.001	1.43 (1.27–1.62)	<0.001
**Chronic kidney failure**					
No	6.8 (1,131/16,532)	1	—	1	—
Yes	9.3 (388/4,150)	1.40 (1.25–1.58)	<0.001	1.15 (1.01–1.32)	0.032
**COPD**					
No	7.4 (1,249/16,901)	1	—	NA	—
Yes	7.1 (270/3,781)	0.96 (0.84–1.10)	0.60	NA	—
**Obesity**					
No	7.6 (1,379/18,160)	1	—	1	—
Yes	5.6 (140/2,522)	0.71 (0.59–0.85)	<0.001	0.76 (0.63–0.91)	0.004
**Ischemic heart disease**					
No	7.0 (1,274/18,301)	1	—	—	—
Yes	10.3 (245/2,381)	1.53 (1.32–1.77)	<0.001	1.28 (1.09–1.49)	0.002
**Asthma**					
No	7.6 (1,416/18,534)	1	—	1	—
Yes	4.8 (103/2,148)	0.61 (0.49–0.75)	<0.001	0.76 (0.61–0.94)	0.014
**Neurodegenerative disease**					
No	6.9 (1,300/18,758)	1	—	1	—
Yes	11.4 (219/1,924)	1.72 (1.49–2.00)	<0.001	1.60 (1.36–1.88)	<0.001
**Solid neoplasia**					
No	6.9 (1,334/19,396)	1	—	1	—
Yes	14.4 (185/1,286)	2.27 (1.92–2.68)	<0.001	2.90 (2.43–3.47)	<0.001
**Lymphoma**					
No	7.3 (1,423/19,496)	1	—	NA	—
Yes	8,1 (96/1,186)	1.11 (0.90–1.39)	0.308	NA	—
**Transplantation**					
No	7.5 (1,492/19,984)	1	—	1	—
Yes	3,9 (27/698)	0.49 (0.33–0.73)	<0.001	0.84 (0.55–1.27)	0.43
**Leukemia**					
No	7.3 (1,485/20,405)			1	
Yes	12.3 (34/277)	1.78 (1.24–2.56)	0.002	3.31 (2.11–4.59)	<0.001
**Cirrhosis**					
No	7.3 (1,496/20,440)	1	—	NA	—
Yes	9.5 (23/242)	1.33 (0.86–2.05)	0.211	NA	—
**HIV infection**					
No	7.4 (1,513/20,484)	1	—	1	—
Yes	3.0 (6/198)	0.39 (0.18–0.88)	0.019	0.74 (0.32–1.72)	0.49
**COVID-19**					
No	7.3 (1,503/20,578)	1	—	1	—
Yes	15.4 (16/104)	2.30 (1.35–3.94)	0.002	1.45 (0.80–2.62)	0.22
**ICU admission**					
No	6.4 (1,226/19,147)	1	—	1	—
Yes	21.7 (284/1,306)	4,06 (3.54–4.68)	<0.001	6.23 (5.30–7.33)	<0.001

aOR: adjusted odds ratio; CI: confidence interval; COPD: chronic obstructive pulmonary disease; ICU: intensive care unit; NA: not applicable (not included in model); OR: odds ratio; RSV: respiratory syncytial virus.

^*^Multivariable logistic regression analysis was used to identify independent predictors of ICU admission; model included variables yielding a p-value of less than 0.05 in the crude analysis, plus sex, group of age, and epidemiological season.

^†^The results are presented as percentages (number of events/sample size).

^‡^Adjusted odds ratios were calculated using a multivariate analysis that included age group, epidemiological season, and variables with a p-value <0.05 in the univariate analysis.

Table 4 compares the epidemiological and clinical characteristics of patients who survived vs. those who died during hospitalization due to RSV infection. In the multivariable analysis, age emerged as a significant predictor of in-hospital mortality. Compared with the reference group (15–64 years), an increased risk of death was observed in patients aged 65–79 years (aOR 1.67, 95% CI 1.37–2.04) and especially in those aged over 80 years (aOR 3.32, 95% CI 2.73–4.04). Other independent risk factors for IHM included ICU admission, leukemia, neoplasms, RSV-related pneumonia, neurodegenerative disease, chronic heart failure, ischemic heart disease and chronic kidney disease Additionally, the epidemiological seasons around the pandemic period, from 2019–2020 to 2021–2022, were independently associated with a higher risk of IHM. Conversely, obesity and asthma were associated with a lower risk. The predictive model demonstrated good discriminative ability, with an AUC of 0.723 (95% CI 0.711–0.736, *p* < 0.001).

## 4 Discussion

This study describes the characteristics and severity of RSV infection hospitalizations in adults over seven epidemiological seasons. Our results show a rising burden of RSV-related hospitalizations over time and highlighted the impact of key risk factors like advanced age, underlying comorbidities, and co-infection with COVID-19 on hospital outcomes such as ICU admission, LOS and in-hospital mortality.

In the first half of the study period, the number of registered cases nearly doubled, from 2658 in 2016–2017 to 5,227 in 2018–2019. This was followed by a precipitous drop (*n* = 50) during the 2020–2021 season, coinciding with the COVID-19 pandemic, and a partial rebound in 2021–2022. From 2016–2017, one factor influencing the estimate of RSV-related hospitalizations has been the increasingly routine use of molecular diagnostic panels, which in Spain accelerated from 2017–18 and after COVID-19 (5). There is still room for improvement in this area, however; in Norway's registry-based surveillance of severe acute respiratory infections from 2021 to 2024, of 214,730 cases, only 53% were tested for RSV—compared to 82% and 73% for SARS-CoV-2 and influenza, respectively (13). Similarly, in Spain, the expanded use of molecular testing has markedly improved RSV detection and contributed to a more accurate assessment of its burden. In Asturias, systematic implementation of molecular techniques (2017–2023) enabled the analysis of over 190,000 respiratory samples, enhancing case detection and genotypic characterization. In Catalonia, the integration of molecular RSV testing into the regional surveillance system also revealed a post-pandemic rebound in RSV activity following the relaxation of COVID-19 control measures (14, 15).

The progressive increase in RSV hospitalizations observed from 2016–2017 to 2018–2019 likely reflects a combination of factors, including improved availability and implementation of molecular diagnostic techniques, increased clinical awareness, and possible true increases in viral circulation. In contrast, the dramatic decline during the 2020–2021 season coincided with the widespread implementation of non-pharmacological interventions aimed at controlling SARS-CoV-2 transmission, including mobility restrictions, lockdown measures, school closures, mask mandates, and enhanced hygiene practices. Similar declines were observed for other respiratory viruses such as influenza (16–18).

The rebound in 2021–2022 can be explained by the relaxation of these preventive measures and the accumulation of a larger susceptible population due to reduced RSV exposure during the pandemic period. This “immunity gap” phenomenon has been described in several countries and likely contributed to the resurgence of RSV activity. Additionally, sustained molecular surveillance in Spain may have facilitated a more accurate detection of post-pandemic RSV cases (14, 15).

Regarding clinical presentation, while bronchitis and other respiratory infections were the most common manifestations in our cohort, nearly a quarter of the total cases presented pneumonia. The association between RSV and pneumonia in adults has been recently highlighted in a German study, in which 3.7% of radiologically confirmed community-acquired pneumonia cases tested positive for RSV, increasing to 7.8% after adjustment for underdetection (19).

Similarly to other studies (13, 18, 20), RSV-related hospitalizations were more frequent in women. It was specially in patients those 80 years older. So in Spain the women generally have a longer life expectancy, and the percentage of admission in 80 years and older is higher in women (21).

In our study, despite fluctuations in incidence, the median length of hospital stay remained stable at approximately 7 days throughout the study period and was associated with comorbidities, ICU admission, and mortality. Overall, RSV-related hospitalizations accounted for nearly 193,873 hospital bed-days, with an estimated annual burden ranging from 467 hospital-days in 2020–2021 to 57,277 in 2018–2019. These figures, reinforced by others published elsewhere in Europe (2–8), underline the substantial impact of RSV on healthcare systems and the need for effective prevention and management strategies, particularly in high-risk populations (1).

Comorbidities play a crucial role in RSV-related hospitalizations. The most frequently observed conditions in our study included hypertension, diabetes, chronic heart failure, chronic kidney disease, COPD, obesity, and asthma. Similar findings were reported in an Ontario cohort (2010–2017), where nearly all hospitalized adults had at least one underlying condition (22). Cardiovascular diseases were present in 35.4% of cases, chronic pulmonary conditions (COPD 44.7%, asthma 32.2%) were common, and 38.4% of patients had immunocompromising conditions (22). In another large cohort, which included 21,250 RSV-related hospitalizations over 12 seasons (2011–12 to 2022–23), 30% had COPD, 29.6% diabetes, 23.4% cardiovascular disease, and 16.8% an immunocompromising condition (23). Loubert et al. (7) also described the relationship between comorbidities and RSV-related hospitalizations and deaths in older patients. Taken together, these data highlight the vulnerability of patients with chronic conditions to severe RSV infection. In addition to the increased risk of complications, extended length of stay in RSV-related hospitalizations represents a major component of healthcare burden and resource utilization. Recognizing predictors of prolonged admission may help optimize inpatient management, anticipate resource allocation, and improve preventive care for high-risk populations.

Like LOS in general wards, ICU admission remained stable over the years and was closely linked to patient age. The ICU admission rate in our cohort was 6.4%, lower than in other published series, which range from 10% in a large cohort across 12 seasons (6,314 hospitalizations) (10), to 13.7% in a Canadian cohort of adults over 50 years (22), and up to 25% in a multinational meta-analysis (24). These differences may be influenced by hospitalization criteria, pneumonia prevalence, and, most importantly, patient characteristics such as age and comorbidities. Admissions to the ICU declined with age, from 12.0% in adults aged under 65 years, to 9.2% in those aged 65–79 years, and only 2.2% in people over 80. This trend aligns with findings from other cohorts, such as Harvers et al.'s (20) in the USA, where ICU admission rates decreased from 21% in patients aged 18–49 years to 16% in those over 75 years. This finding does not necessarily reflect a lower severity of RSV infection in the older adult but rather differences in clinical management. The lower ICU admission rates observed among older adults likely result from triage and clinical decisions to limit invasive therapeutic interventions in frail patients, rather than from lower illness severity. It suggests that most deaths in very old patients occurred outside the ICU, likely due to comorbidities, frailty, and physician decisions to limit invasive therapeutic efforts (25).

The overall mortality rate among hospitalized patients varied by epidemiological season, ranging from 6.1% in 2016–2017 to 18.0% during the COVID-19 pandemic. The global case fatality rate was 7.3%, increasing from 4.0% in patients under 65 years to 9.5% in those aged over 80 years. These findings are consistent with other studies, where in-hospital case fatality rates for RSV range from 5% to 10% and increase with age (2, 23). A systematic review by Nguyen et al. (24) reported an overall RSV-related case fatality rate of 8.2% among adults over 60 years.

The main risk factors for mortality included ICU admission, advanced age, and comorbidities like leukemia, cancer, neurodegenerative diseases, heart failure, and chronic kidney disease. As observed in all cohorts, age and comorbidities are consistently associated with mortality in adults hospitalized for RSV (2, 7, 25).

Currently, treatment for RSV infection is mainly supportive, as no RSV-specific antiviral therapy is available. Ribavirin has been used in selected severe cases, although its efficacy remains limited (26). Palivizumab, a monoclonal antibody, is used as prophylaxis in high-risk infants (26). Consequently, prevention remains one of the most effective strategies to reduce RSV-associated morbidity and mortality. A recent meta-analysis evaluating three RSV vaccines demonstrated a pooled efficacy of 81.3% for preventing lower respiratory tract disease with three or more signs or symptoms during the first RSV season (27).

Given the strong association between age, comorbidities, and RSV-related mortality, in 2024 the CDC Advisory Committee on Immunization Practices (ACIP) recommended RSV vaccination for all individuals over 75 years of age as well as for those aged 60–75 with chronic heart or lung diseases, frailty, or those residing in long-term care facilities (28).

In our study, asthma and obesity appeared to be protective factors against in-hospital mortality among patients admitted with RSV infection. This finding may be partly explained by the younger age, lower comorbidity, and closer medical monitoring of patients with asthma, as previously reported in a national study showing no increased mortality among older adults hospitalized with RSV and asthma (29). The apparent protective effect of obesity may reflect the “obesity paradox” described in respiratory and infectious diseases such as pneumonia and sepsis, where greater metabolic reserves, low-grade inflammation, and earlier medical attention could improve short-term outcomes (30). Nevertheless, these results should be interpreted with caution due to potential residual confounding and limitations of administrative data, including coding bias and lack of detailed severity information.

Our study has several strengths. First, we used a medical-administrative database with coding rules that included all RSV-related hospitalizations in adults across Spain over a seven-season period. Second, to our knowledge, our study is the first to analyze RSV hospitalization in adults and mortality during the COVID-19 pandemic. Third, the study adopted a multidisciplinary approach, incorporating epidemiological data and clinical data to identify risk factors for LOS, ICU admission, and IHM.

Limitations include the study's retrospective nature, precluding a review of patients' medical histories, which would have allowed us to verify data accuracy (quality of information on causes of death). In addition, the medical administrative database lacked laboratory or clinical test results, as well as details on the treatments administered, vaccination history, or laboratory-confirmed viral co-infections. Consequently, the potential impact of these factors on the three study endpoints—ICU admission, prolonged length of stay, and in-hospital mortality—could not be evaluated. There are also limitations inherent to the use of medical-administrative databases with coding rules. We included only cases coded with one of the four specific ICD-10 codes for RSV (J21.0, J12.1, J20.5, and B97.4), assuming these cases were diagnosed using molecular procedures. Nevertheless, specific laboratory confirmation for RSV was not available in the dataset. Therefore, we did not analyze hospitalization rates by population or admissions, and the number of RSV-related cases is likely underestimated. In contrast, other studies have included a broader range of ICD-10 codes not specifically tied to RSV to represent RSV-associated respiratory illnesses, including J20.9 (acute bronchitis, unspecified), J21.9 (acute bronchiolitis, unspecified), J40 (bronchitis, not specified as acute or chronic), and J45.9 (other and unspecified asthma) (8, 9). Our approach enhances specificity by focusing solely on RSV-associated episodes, but excludes patients admitted for other clinical conditions caused by RSV but without molecular confirmation. As a result, our findings are highly specific to RSV but less sensitive, potentially missing cases where RSV was the underlying cause of hospitalization but was coded differently.

## 5 Conclusion

The incidence of RSV-related hospitalizations in adults has been increasing over time, with a temporary decline during the COVID-19 pandemic. While this trend may be partially influenced by increased diagnostic efforts in patients hospitalized for respiratory infections, it reflects the significant burden RSV poses to healthcare systems. The most affected groups are older adults and people with multimorbidity, with an estimated in-hospital mortality rate of approximately 6%. RSV thus represents a growing public health concern. Expanding RSV vaccination to individuals over 60 years of age with underlying health conditions could be an effective measure to further reduce RSV-related morbidity and mortality.

## Data Availability

The raw data supporting the conclusions of this article will be made available by the authors, without undue reservation.
