# Supplementary material for: Adult RSV hospitalizations in Spain: clinical characteristics and risk factors for ICU admission, prolonged stay, and mortality across six seasons (2016–2017 to 2021–2022)
Source: Front Public Health. 2026 Mar 16;14:1781582. doi: 10.3389/fpubh.2026.1781582 (PMC13033660; doi:10.3389/fpubh.2026.1781582)
Supplement: Supplementary file 1 [file Table_1.docx]

**Supplementary Tables.**

**Supplementary Table 1. International Classification of Diseases, 10th edition codes used in the study.**

| **Diagnosis** | Code |
| --- | --- |
| **Pneumonia due to respiratory syncytial virus** | J12.1 |
| **Acute bronchitis due to respiratory syncytial virus.** | J20.5 |
| **Acute bronchiolitis due to respiratory syncytial virus** | J21.0 |
| **Respiratory syncytial virus as the cause of diseases classified elsewhere** | B97.4 |
| **Comorbidities** |  |
| **Hypertension** | I10; I11; I12; I13; I15 |
| **Diabetes mellitus** | E10.x; E11.x; E13.x |
| **Neoplasms** | C01; C02; C03; C04; C05; C06; C07; C08; C09; C10; C11; C12; C13; C14; C15; C16; C17; C18; C19; C20; C21; C22; C23; C24; C25; C26; C27; C28; C29; C30; C31; C32; C33; C34; C35; C36; C37; C38; C39; C40; C41; C42; C43; C44; C45; C46; C47; C48; C49; C50; C51; C52; C53; C54; C55; C56; C57; C58; C59; C60; C61; C62; C63; C64; C65; C66; C67; C68; C69; C70; C71; C72; C73; C74; C75; C76; C77; C78; C79; C80 |
| **Obesity** | E66.x; Z68.3; Z68.4 |
| **Chronic kidney disease** | N18.x |
| **Ischemic heart disease** | I21.x; I22.x; I23.x; I24.x; I25.x |
| **COPD** | J44.x |
| **Chronic Heart failure** | I50.x |
| **Leukemia** | C92; C93; C94; C95; C96 |
| **Lymphoma** | C81; C82; C83; C84; C85; C86 ; C87 ; C88 ; C89 ; C90; C91 |
| **Cirrhosis** | K74.x; K70.3 |
| **HIV** | B20.x |
| **Neurodegenerative disease** | F01.x; F02.x; F03.x; G30.x; G31.x; G32.x |
| **Transplantation** | Z94.x |
| **COVID-19** | B97.4 |
